# Arterial cerebrovascular complications in 94 adults with acute bacterial meningitis

**DOI:** 10.1186/cc10565

**Published:** 2011-11-23

**Authors:** Matthias Klein, Uwe Koedel, Thomas Pfefferkorn, Grete Zeller, Bianca Woehrl, Hans-Walter Pfister

**Affiliations:** 1 Department of Neurology, Klinikum Grosshadern, Ludwig Maximilians University, Marchioninistr. 15, D-81377 Munich, Germany

## Abstract

**Introduction:**

Intracranial vascular complications are an important complication of acute bacterial meningitis. Ischemic stroke in meningitis is reported as a result of vasculitis, vasospasm, endocarditis or intraarterial thrombosis. The aim of the study was to identify the value of measuring cerebral blood flow velocity (CBFv) on transracranial doppler (TCD) in the identification of patients at risk for meningitis-associated stroke.

**Methods:**

We retrospectively studied patients with acute bacterial meningitis who were treated in our university hospital from 2000 to 2009. Data were analyzed with the main focus on the incidence of an increase of CBFv on TCD, defined as peak systolic values above 150 cm/s, and the development of stroke.

**Results:**

In total, 114 patients with acute bacterial meningitis were treated, 94 of them received routine TCD studies during their hospital stay. 41/94 patients had elevated CBFv values. This increase was associated with an increased risk of stroke (odds ratio (95% confidence intervall) = 9.15 (1.96-42.67); p < 0.001) and unfavorable outcome (Glasgow Outcome Score < 4; odds ratio (95% confidence intervall) = 2.93 (1.23-6.98); p = 0.018). 11/32 (34.4%) patients with an increase of CBFv who received nimodipine and 2/9 (22.2%) patients with an increase of CBFv who did not receive nimodipine developed stroke (p = 0.69).

**Conclusions:**

In summary, TCD was found to be a valuable bedside test to detect arterial alterations in patients with bacterial meningitis. These patients have an increased risk of stroke.

## Introduction

The development of cerebrovascular alterations is an important intracranial complication in acute bacterial meningitis and is associated with poor outcome [1]. Arterial cerebrovascular complications are reported to occur in approximately one-fifth of patients [2]. Using digital subtraction angiography (DSA), arterial narrowing is the predominant finding in patients with arterial complications, involving all vessel sizes [1]. The underlying reason for narrowing of the cerebral arteries during bacterial meningitis is still a matter of debate. Autopsy and animal model studies indicate severe inflammation of the vessel walls (vasculitis) as a key etiology [3,4]. Furthermore, patients in whom histopathological correlates in terms of inflammation were not found at the sites of arterial narrowing have been reported [5,6]. This suggests vasospasm as a second important etiology. Also, ischemic stroke can occur as the result of endocarditis and septic emboli. Recently, the development of arterial cerebral infarctions was reported to occur up to weeks after initial recovery from pneumococcal meningitis and an association with adjunctive steroid therapy was suggested [7]. Autopsy studies in such patients showed intraarterial clotting but no signs of vasculitis and intraarterial thrombosis was suggested to be a fourth reason for the development of stroke [7]. Furthermore, diffuse cerebral vascular coagulation was proposed as the main etiology for stroke in another autopsy series on 15 patients who died from pneumococcal meningitis [8]. Unfortunately, *in vivo *vascular imaging was not performed in these two studies. Given the fact that arterial complications in bacterial meningitis are associated with a poor prognosis, the question how arterial alterations can be detected early and in a feasible way during bacterial meningitis arises. Here, we retrospectively studied the data of 114 patients with acute bacterial meningitis who were treated at our university hospital from 2000 to 2009 with the scope on an increase of cerebral blood flow velocity (CBFv) on transcranial Doppler ultrasound (TCD) and its link with stroke and an adverse outcome.

## Materials and methods

### Patients

Data of all patients with acute bacterial meningitis who were treated at the Department of Neurology at Klinikum Grosshadern, the teaching hospital of the Ludwig-Maximilians University Munich, Germany, from 2000 until 2009 were analyzed. Bacterial meningitis was diagnosed through (i) isolation of bacteria from the cerebrospinal fluid (CSF) and/or (ii) the presence of clinical symptoms (fever, headache, impaired consciousness, and/or neck stiffness) in combination with typical alterations of the CSF. Typical CSF findings were defined as a mainly granulocytic CSF white blood cell count (CSF-WBC) of more than 1,000 cells/μl, an increase of CSF protein of more than 100 mg/dl, and a CSF/serum glucose ratio less than 0.3.

### Detection of arterial narrowing

The presence of vascular alterations was assessed by TCD of the anterior cerebral artery (ACA), middle cerebral artery (MCA), internal cerebral artery (ICA), posterior cerebral artery (PCA), and the basilar artery (BA). Systolic CBFv greater than 150 cm/s were considered increased [9].

### Stroke and outcome in patients with arterial narrowing

Arterial vascular alterations and the incidence of cerebral ischemia were evaluated using magnetic resonance imaging (MRI) and computed tomography (CT) [10]. Only lesions with a configuration typical of ischemic stroke (corresponding to an arterial vessel territory or watershade infarctions) were diagnosed as cerebral ischemia. On MRI, ischemic stroke was diagnosed when lesions were found to be hyperintense on diffusion-weighted imaging and T2-weighted imaging. On CT scan, ischemic lesions appeared hypodense. Effects of arterial vascular alterations on outcome measured with the Glasgow Outcome Scale (GOS; 1 = death, 2 = vegetative state, 3 = severe disabilities, 4 = moderate disabilities, 5 = no/mild disabilities) were assessed.

### Statistical analysis

Systat 9 (Chicago, IL, USA) was used for statistical analysis. The Wilcoxon-Mann-Whitney test and Chi squared test as well as the Fisher's exact test was used for analysis of demographic and clinical characteristics of patients with and without vascular alterations. Odds ratios and 95% confidence intervals were calculated. *P *< 0.05 was considered significant. The study is in compliance with the Declaration of Helsinki on Ethical Principles for Medical Research involving Human Subjects. Due to the completely retrospective nature of the study without the possibility to identify patients from the anonymous collected data, the ethics committee of the University of Munich gave ethical approval and waved the need for informed consent of the patients.

## Results

### Patients

From 2000 to 2009, 114 adult patients with bacterial meningitis were treated in our hospital. A causative pathogen was identified in 95 cases (83%). The most common pathogens were *Streptococcus pneumoniae *(48 of 95 patients; 50.5%) followed by *Staphylococcus aureus *(10 of 95 patients; 10.5%) and *Neisseria meningitidis *(9 of 95 patients; 9.4%). Overall mortality was 15%.

### Vascular complications

In 94 of 114 patients, data on TCD studies were available. High systolic CBFv greater than 150 cm/s were detected in 41 of 94 (43%) patients (systolic CBFv ± standard deviation = 222 ± 45 cm/s). In 63% of these patients, CBFv was increased in vessels of both the anterior and posterior circulation. In 35% of patients, the increase of CBFv was noted only in the anterior circulation, and in 2% only in the posterior circulation. An increase of CBFv on TCD was detected in 45% of patients within two days after treatment was begun. In 21% of patients, changes were detected more than seven days after initiation of therapy. In one patient, changes were observed until 51 days after the start of therapy. However, this particular patient suffered from subarachnoid hemorrhage as an alternative reason for an increase of CBFv. In 6 of 13 patients who received arterial MRI, vascular alterations that were found on TCD were confirmed by MR angiography. Vascular alterations were also confirmed in one of five patients who received CT angiography and in two of two patients who received DSA. One of these patients also received MR and CT angiography, revealing no vascular alterations; the other one received MR angiography and DSA, both showing arterial narrowing.

### Factors associated with an elevation of CBFv

The occurrence of vascular alterations on TCD was associated with a lower Glasgow Coma Score (GCS) on admission (Table 1). TCD alterations were not associated with certain causative pathogens (Table 1). CSF WBC, CSF albumin content, CSF glucose, and CSF/serum glucose on admission were not different in patients with and without increased CBFv on TCD. Data on the application of corticosteroids was available in 82 patients. Patients who showed increased CBFv on TCD had more often received corticosteroids as adjunctive therapy on admission to the hospital (Table 1). The group of patients who received corticosteroids during initial therapy presented with a lower GCS on admission (*P *= 0.002).

**Table 1:** Risk factors for increased sCBF in patients with bacterial meningitis

	All patients	Increase in CBFv	No increase in CBFv	Odds ratio (95% CI)	*P *value
	*n *= 94	*n *= 41 (43.6%)	*n *= 53 (56.4%)		
**Male/Female**	38/56	15/26	23/30	0.75 (0.32-1.73)	0.53
**Age in years**	56(16-81)	46(18-81)	59(16-81)		0.125
**GCS (admission)**	11(3-15)	11(3-15)	12(7-15)		*0.033*
**Causative pathogen**					
***Streptococcus pneumoniae***	46	23 (56.1%)	23 (43.4%)	1.67 (0.73-3.79)	0.218
***Neisseria meningitidis***	8	4 (9,7%)	4 (7.5%)	1.05 (0.25-4.52)	0.725
***Staphylococcus aureus***	6	2 (4.9%)	4 (7.5%)	0.62 (0.11-3.61)	0.693
***Haemophilus influenzae***	4	1 (2.4%)	3 (5.7%)	0.42 (0.04-4.16)	0.629
***Streptococcus intermedius***	3	1 (2.4%)	2 (3.7%)	0.69 (0.06-7.89)	1.00
Others*	12	5 (12.2%)	7 (13.2%)	0.91 (0.27-3.12)	1.00
**Pathogen not detected**	17	8 (19.5%)	9 (17.0%)	1.45 (0.51-4.14)	0.792
***CSF findings on admission***					
**CSF cell count (cells/μl)**	1600 (7-37800)	986 (7-10667)	2208 (35-37800)		0.374
**CSF albumin (mg/dl)**	254 (39-4455)	236 (39-1358)	277 (52-4455)		0.677
**CSF glucose (mg/dl)**	16 (0-175)	12 (0-64)	23 (0-175)		0.256
**CSF/serum glucose**	0.19 (0.00-0.59)	0.14 (0.00-0.45)	0.21 (0.02-0.59)		0.655
**Adjunctive therapy with corticosteroids**	34/82	20/36	14/46	*2.86 (1.15-7.09)*	*0.026*

(*) *Streptococcus mitis, Streptococcus pyogenens, Listeria monocytogenes, Streptococcus agalacticae, Acinetobacter baumanii, Fusobacterium necrophorum, Escherichia coli*. Multiple pathogens were detected in three patients.

(#) In one patient, Gram-positive bacteria were seen on Gram stain but a further differentiation was not successful.

CBFv, cerebral blood flow velocity; CI, confidence interval; CSF, cerebrospinal fluid; GCS, Glasgow Coma Scale.

### Increased CBFv and outcome

Increased CBFv was associated with an odds ratio of 9.15 (95% confidence interval: 1.96 to 42.67) for the development of ischemic stroke. Ischemic stroke was found in 14 of 41 patients with TCD alterations (two of them were also suffering from endocarditis as a possible source of embolism), whereas only 2 of 51 patients without TCD alterations suffered from stroke (Table 2). In 3 of the 14 patients with increased CBFv, stroke was not restricted to the vessel territory in which increased CBFv were detected (one of them suffering from endocarditis). In the group of patients with stroke but no TCD alterations, one suffered from atrial fibrillation as a possible etiology of cerebral ischemia. In the other patient, a possible etiology was not found. If stroke occurred in more than one arterial territory, an increase of CBFv in all vessels and/or endocarditis was detected (Table 3). An increase of CBFv was associated with unfavorable outcome (GOS 1, 2, or 3; Table 2). 32 patients with an increase of CBFv received therapy with nimodipine (intravenously or orally), 11 of them (34.4%) developing stroke (2 of the 11 patients suffering from endocarditis). Two of nine patients (22.2%) with an increase of CBFv who did not receive nimodipine experienced cerebral ischemia (*P *= 0.69).

**Table 2:** An increase of rCBF is associated with stroke and poor outcome

	All patients	Increase in CBFv	No increase in CBFv	Odds Ratio (95% CI)	*P *value
**Ischemic stroke**	16	14	2	*9.15 (1.96-42.67)*	*< 0.001*
Glasgow Outcome Scale (GOS)*	4 (1-5)	3 (1-5)	4 (1-5)		*0.029*
**GOS 1, 2 or 3**	37	22	15	*2.93 (1.23-6.98)*	*0.018*
**GOS 4 or 5**	54	18	36	*0.34 (0.14-0.81)*	*0.018*
**Time until discharge from ICU (days)**	12 (1-112)	15 (3-112)	9 (1-59)		0.096

*Data available for 91 patients.

CBFv, cerebral blood flow velocity; CI, confidence interval.

**Table 3:** Characteristics of patients with meningitis and stroke

	Age	Pathogen	GCS	GOS	TCD			Stroke			Concomitant complications	Remarks
					Time point	CBFv	Location	Time point	Imaging	Location		
	**years**		**3-15**	**1-5**	**days**	**cm/s**		**days**				

1	79	*S- pneum*.	7	1	10	> 150	Basilar artery	12	MRI	Posterior circulation	Hydrocephalus, epileptic seizures, sepsis	Arterial narrowing on MRA
2	67	*S. pneum*.	3	1	12	300	All vessels	12	MRI	MCA, ACA, posterior circulation	Hydrocephalus, epileptic seizures, severe sepsis, subarachnoid hemorrhage	Arterial narrowing on digital subtraction angiography
3	27	N/A	5	1	1	> 150	posterior circulation	1	CT	left MCA and ACA, posterior circulation	Severe sepsis	
4	56	*S. pneum*.	9	2	51	> 150	N/A	51	MRI	posterior circulation	Hydrocephalus	Arterial narrowing on MRA
5	62	*S. pneum*.	7	2		250	All vessels	16	MRI	right MCA, posterior circulation	Hydrocephalus, empyema, paravertebral abscesses	
6	65	*S. pneum*.	3	2	5	300	All vessels	21	MRI	right MCA	Hydrocephalus epileptic seizures, sepsis	
7	43	*S. pneum*.	9	2	7	180	All vessels	5	MRI	left ACA, left MCA, posterior circulation	subarachnoid hemorrhage	Arterial narrowing on digital subtraction angiography, Increased CBFv in TCD documented for 2 months
8	44	*S. pneum*.	5	3	0	210	All vessels		CT	left MCA (watershade)	Hydrocephalus, bilateral hypakusis	Arterial narrowing on MRA
9	40	*S. pneum*.	12	3	9	270	All vessels	17	MRI	Posterior circulation	Hydrocephalus, cerebritis	
10	77	N/A	12	3	23	258	Right and left MCA, posterior circulation	N/A	MRI	Posterior circulation	Intracranial hemorrhage	
11	37	Gram-positive bacteria*	6	3	25	> 150	All vessels	17	MRI	Posterior circulation	Brain oedema, sepsis, ARDS, renal failure	
12	64	*E. coli*	11	3	N/A	> 150	All vessels	1	MRI	left MCA		Endocarditis
13	37	*S. pneum*.	7	4	1	230	All vessels	18	MRI	Right and left MCA	Hydrocephalus, Hypakusis	
14	42	*S. agalacticae*	11	N/A	0	230	Right and left MCA	1	MRI	Left ACA and MCA		Endocarditis
15	67	*S. pneum*.	7	4	10	< 150	All vessels	9	MRI	Right and left ACA		
16	76	*Pseud. aeruginosa*	9	4	0	< 150	All vessels	0	MRI	Posterior circulation, left MCA		atrial fibrillation

(*) On Gram stain, further differentiation not possible. ACA, anterior cerebral artery; CBFv, cerebral blood flow velocity; *E. coli, Escherichia coli*; GCS, Glasgow Coma Scale; GOS, Glasgow Coma Scale; MCA, medial cerebral artery; MRI, magnetic resonance imaging; *Pseud., Pseudomonas; S. agalacticae, Streptococcus agalacticae; S. pneum., Streptococcus pneumonia*; TCD, transcranial Doppler.

### Findings on autopsy

In one patient with increased CBFv, corresponding arterial narrowing on DSA, and cerebral ischemia, inflammation was seen surrounding middle and small sized blood vessels on autopsy as a possible underlying reason for the vascular alterations found *in vivo*. Autopsy data from other patients were not available (due to denial of autopsy by relatives).

## Discussion

The major findings of our study were that (i) 43.6% of patients showed vascular alterations on TCD and (ii) these alterations were seen more often in patients with a poor GCS on admission. Increased systolic CBFv greater than 150 cm/s evidenced by TCD were associated with (iii) the development of ischemic stroke and (iv) unfavorable outcome. (v) Treatment of an increase of CBFv with nimodipine was not associated with a reduction of stroke.

The current study demonstrates that an increase of CBFv is common in bacterial meningitis. Here, we detected an increase of systolic CBFv greater than 150 cm/s in 41 of 94 patients (43.6%) with acute bacterial meningitis. In earlier smaller case series, similar rates have been reported: for example, an increase of the CBFv was found in 23 of 41 patients (51%) with acute bacterial meningitis [11] and in 27 of 53 (51%) patients with bacterial meningitis during the first two weeks of admission [12]. Also, a very high incidence of vascular alterations was reported in a case series of 22 patients with bacterial meningitis, with markedly increased systolic peak velocities (> 210 cm/s) in seven patients [13]. Taken together with the high incidence of TCD alterations found in the large group of patients of this study, this demonstrates that cerebrovascular complications are frequently found in patients with bacterial meningitis. The high incidence of cerebrovascular complications found on TCD, in comparison with that reported by studies looking at alterations on MR angiography, DSA, and ischemic stroke in selected patients, is likely to only be related to the simplicity of using TCD. This also allowed screening for vascular alterations in critically ill patients without the necessity for invasive, time consuming, and, sometimes, risky investigations that are often only performed if focal neurologic signs appear. In addition, the cost is low. One limitation of TCD is the necessity to obtain bone cranial windows, which might be difficult in some patients. However, the use of bedside TCD seems to be a helpful and feasible tool for clinical practice to screen patients with bacterial meningitis for vascular alterations, aiming at the identification of patients at high risk for ischemic stroke.

Alterations of CBFv on TCD were seen more often in patients that were severely affected clinically on admission. This is in line with the previous observations that a low GCS and a low CSF glucose on admission are risk factors for intracranial complications and poor outcome [2,14]. Calculation of odds ratios identified initial adjunctive corticosteroid treatment to be associated with the development of arterial complications. One likely explanation is that corticosteroids were especially given to patients with severe clinical affection on admission. Thus, it cannot be concluded from the results of this study that adjunctive corticosteroids lead to the development of arterial narrowing. However, it has to be kept in mind that case series discussing a possible association of steroid use and stroke in pneumococcal meningitis have recently been published [7].

An increase of CBFv greater than 150 cm/s on TCD was associated with an increased incidence of ischemic stroke. Nevertheless, negative TCD studies do not rule out the chance of ischemic stroke: 2 of 53 meningitis patients without an increase of CBFv on TCD developed cerebral ischemia (one of them suffering from endocarditis). In the daily routine on our ICU, systolic CBFv greater than 150 cm/s was considered indicative of vasculopathy. Nevertheless, arterial narrowing indicated by an increase of CBFv was only confirmed by MR angiography in 6 of 13 patients, CT angiography in one of five patients, and DSA in two of two patients. Unfortunately, exact timely correlations with imaging studies, especially DSA (gold standard) were not possible from our data. Thus, it remains unclear which threshold of CBFv on TCD should be considered pathologic in patients with bacterial meningitis. Nevertheless, here, a threshold of 150 cm/s helped to identify 14 of 16 patients (87.5%) with stroke.

Vascular alterations occur mainly during the first days after admission but can be found even later. If alterations were detected on TCD in this study, they were present more than one week after admission in 21%. This is in line with the observation that stroke can occur later after the onset of bacterial meningitis [1,3,7,15]. As this was a retrospective study without a predetermined time point of TCD and/or other imaging evaluation of the patient, the exact onset of vascular alterations and/or stroke might have been missed. Nevertheless, the fact that an increase in CBFv was closely associated with stroke in such a high percentage even though TCD was performed not on a predetermined timely basis underlines its value in clinical practice. Altogether, this demonstrates the necessity to monitor patients early, closely, and long enough in order to identify patients at risk of stroke early.

What can be done if vascular alterations such as arterial narrowing are detected? An increase of CBFv on TCD can be the result of narrowing of the arterial lumen or hyperperfusion. Possible underlying reasons for narrowing of cerebral arteries in meningitis were shown to be vasospasm and/or vasculitis, as evidenced by histopathology in patients who died during acute bacterial meningitis [1,3]. Clearly, randomized controlled trials on possible treatment strategies of arterial narrowing in bacterial meningitis are non-existent. Another disease with prolonged narrowing of cerebral arteries is subarachnoid hemorrhage (SAH) [16], TCD having become a valuable tool to identify patients with SAH-associated vasospasms [17,18]. Such patients with SAH that develop cerebral vasospasms are often treated with an elevation of blood pressure, hypervolemia, and hyperperfusion (triple H therapy) in combination with intravenous or oral nimodipine [19]. Whether such therapeutic strategies can be transferred to practice in bacterial meningitis is unclear at present. Due to the risk of cerebral bleeding in infections of the central nervous system, extensive hypertension might even be harmful to the patient. Nimodipine would potentially also act in the case of meningitis-associated vasospasm (but not vasculitis) as the underlying etiology for an increase of CBFv on TCD. Here, therapy with intravenous or oral nimodipine was not of benefit. One could argue that a possible effect of nimodipine might have been missed due to the very small number of patients not receiving nimodipine, but the proportion of patients with stroke despite nimodipine treatment was high. Administration of nimodipine is often associated with a drop of the arterial blood pressure. Keeping in mind that the cerebral autoregulation can be affected during bacterial meningitis [20], such a decrease of the blood pressure could easily affect the cerebral perfusion pressure, potentially being of risk to the patient (especially in the case of vasculitis of large and small blood vessels). Combined, nimodipine cannot be recommended for routine therapy of meningitis-related TCD alterations.

Intraarterial clotting might be another mechanism for the development of stroke. Thus, anticoagulation with heparin or antiplatelet strategies could be discussed as potential treatment strategies. However, given the fact that intracranial bleeding is a frequent complication in pneumococcal meningitis, administration of drugs that interfere with coagulation seem dangerous. In a small study on the use of heparin which included 15 patients with bacterial meningitis, outcome not improved in patients receiving heparin vs. those not receiving heparin (mortality 57% vs. 25%, respectively) [21]. Thus, the use of heparin cannot be recommended without clinical studies. If strategies that aim at the inhibition of the coagulation cascade or stimulation of fibrinolysis could be of benefit in bacterial meningitis yet need to be evaluated in experimental studies.

Due to its retrospective nature, this study has several limitations. Only 94 of 114 patients with bacterial meningitis received TCD studies. However, it has to be noted that the outcome of the remaining 20 patients without TCD studies was worse than in the group of patients with TCD studies (mortality in patients without TCD studies = 55% vs. mortality in patients with TCD studies = 5.3%). The major reason for this selection bias was that in many of the non-TCD-investigated patients, clinical problems such as renal failure with the need of dialysis, airway control, and severe sepsis and extensive blood pressure management as well as control of intravascular disseminated coagulopathy dominated the treatment of these patients, giving highest priority to high urgent directly life saving measures. Thus, the actual number of patients with an increase of CBFv on TCD might have been underestimated. Unfortunately, continuous TCD studies were not available in this patient series, therefore we cannot comment on the course of vascular alterations and it is unclear how long patients with vascular alterations remain at risk for ischemia. However, the fact that patients with bacterial meningitis develop stroke even weeks after meningitis shows that a high level of suspicion for vascular complications should be sustained even after the acute stage of the disease is overcome.

## Conclusions

In summary, this study demonstrates that vascular alterations are common in bacterial meningitis. The fact that the development of vascular alterations is associated with stroke and unfavorable outcome underlines its importance for the course of the disease. TCD is shown to be an easy to perform bedside test, not being associated with risks to the patient. Thus, TCD seems an excellent tool to identify patients at high risk for the development of stroke and unfavorable outcome. Clearly, studies on possible treatment strategies of vascular complications in bacterial meningitis are needed.

## Key messages

◆ An increase of CBFv on TCD is common in patients with acute bacterial meningitis.

◆ An increase of CBFv on TCD is associated with ischemic stroke and poor outcome in acute bacterial meningitis.

◆ TCD is a very easy and useful investigation to detect patients at risk for cerebral ischemia in bacterial meningitis.

## Abbreviations

ACA: anterior cerebral artery; BA: basilar artery; CBFv: cerebral blood flow velocity; CSF: cerebrospinal fluid; CT: computed tomography; DSA: digital subtraction angiography; GCS: Glasgow Coma Scale; GOS: Glasgow Outcome Scale; ICA: interal cerebral artery; MCA: middle cerebral artery; MRI: magnetic resonance imaging; PCA: posterior cerebral artery; SAH: subarachnoid hemorrhage; TCD: transcranial doppler sonography; WBC: white cell count.

## Competing interests

The authors declare that they have no competing interests.

## Authors' contributions

MK developed the study design and carried out the data collection, data analysis, manuscript draft, and revision. UK contributed with critical manuscript revisions. TP contributed with critical manuscript revisions. GZ carried out data collection and manuscript revision. BW contributed with manuscript revision. HWP brought up the study idea and carried out critical manuscript revision. All authors have read and approved the manuscript for publication.

## Authors' information

MK, GZ and BW are residents at the Department of Neurology at Munich University with a main focus of their education in neurocritical care. In addition, MK, BW, UK, and HWP have a long history of experimental and clinical research in bacterial meningitis. TP and HWP work as attending at the Department of Neurology at Munich University, HWP is the assistant director of the Department of Neurology at Munich University.
